# Epidemiological study of *HER-2* mutations among EGFR wild-type lung adenocarcinoma patients in China

**DOI:** 10.1186/s12885-016-2875-z

**Published:** 2016-10-28

**Authors:** Xuefei Li, Chao Zhao, Chunxia Su, Shengxiang Ren, Xiaoxia Chen, Caicun Zhou

**Affiliations:** 1 Department of Lung Cancer and Immunology, Shanghai Pulmonary Hospital, Tongji University, No. 507 Zhengmin Road, Shanghai, 200433 People’s Republic of China; 2 Department of Medical Oncology, Shanghai Pulmonary Hospital, Tongji University Medical School Cancer Institute, Tongji University School of Medicine, No. 507 Zhengmin Road, Shanghai, 200433 People’s Republic of China

**Keywords:** HER-2, ALK, ROS1, Lung adenocarcinoma

## Abstract

**Background:**

*Human epidermal growth factor receptor* (*HER*)*-2* is a driver gene in non-small cell lung cancer (NSCLC). The present study evaluated the mutation rate of *HER-2* within the wild-type *epidermal growth factor receptor* (*EGFR*) lung adenocarcinoma population in China.

**Methods:**

Formalin-fixed, paraffin-embedded samples from 456 patients with wild-type EGFR lung adenocarcinoma were analyzed for *HER-2* mutations by amplification-refractory mutation system (ARMS), and HER-2 protein expression was evaluated by immunohistochemistry. All samples positive for *HER-2* mutation underwent direct sequencing for further verification.

**Results:**

*HER-2* mutation was detected in 22/456 cases (4.8 %); the rate was 6.7 % among 331 triple-negative samples (i.e., wild-type EGFR, anaplastic lymphoma kinase, and ROS proto-oncogene 1). Direct sequencing confirmed that the results were consistent with those obtained by ARMS analysis in 19 cases. The positive rate was 15.4 % by immunohistochemical analysis of HER-2 expression; this was not correlated with mutation rate. *HER-2* mutation and positivity were not correlated with gender, age, smoking status, disease stage, or histological subtype. The 22 cases of *HER-2* mutations occurred only in acinar (36.4 %), papillary (36.4 %), minimally invasive (13.6 %), solid (9.2 %), and invasive mucinous (4.5 %) subtypes. Disease-free and overall survival were not associated with *HER-2* mutation or HER-2 protein overexpression.

**Conclusion:**

The *HER-2* mutation rate was 4.8 % among *EGFR* wild-type lung adenocarcinoma patients in China, and 6.7 % among driver genes, triple-negative lung adenocarcinoma. The incidence of *HER-2* mutation varied among different lung adenocarcinoma subtypes, occurring mainly in acinar and papillary predominant subtypes. 15.4 % of *EGFR* wild-type lung adenocarcinoma patients showed HER-2 protein overexpression, but this was not correlated to *HER-2* mutation. Existing follow-up data did not show a correlation between *HER-2* mutation with DFS or OS.

**Electronic supplementary material:**

The online version of this article (doi:10.1186/s12885-016-2875-z) contains supplementary material, which is available to authorized users.

## Background

Human epidermal growth factor receptor (HER)-2 is a member of the epidermal growth factor receptor (EGFR) family, in which it is considered as having the highest activity. HER-2 forms heterodimers with other family members to activate downstream mitogen-activated protein kinase and phosphoinositide 3-kinase/Akt signaling pathways [1–4]. Somatic mutations have been identified within the kinase domain of HER-2 [5]; the *HER-2* mutation rate in non-small cell lung cancer (NSCLC) is 2 %–4 % [6–8]. However, the relationship between *HER-2* mutation and the clinical characteristics and prognosis of lung adenocarcinoma remains unclear, not least because of inconsistencies among findings reported by various studies. For example, some studies have shown that *HER-2* mutations were prevalent among non-smoking Asian females [9–11], while another found that *HER-2* mutation was related to disease stage [12]. HER-2 protein was overexpressed in 13 %–20 % of NSCLC patients [11]; a strong positive expression (i.e., immunohistochemistry score of 3+) was observed in 2 %–6 % of cases [12, 13]. HER-2 overexpression has also been linked to poor patient prognosis [14–16].

The present study investigated the *HER-2* mutation rate in lung adenocarcinoma patients in China with wild-type EGFR and its relationship to clinical characteristics and prognosis.

## Methods

### Patients and study design

**Fig. 1:**
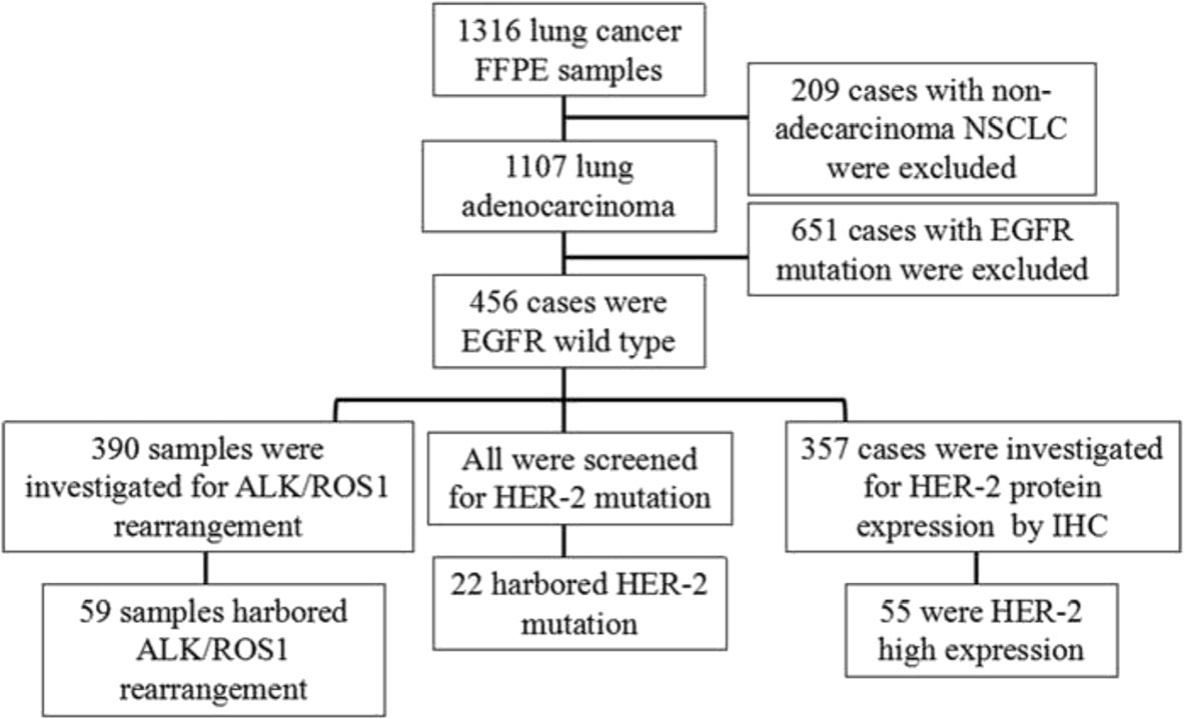
Study design. 456 cases with EGFR wild type were screened from 1316 patients and enrolled in this study

The inclusion criteria were as follows: patients over 18 years old; confirmed diagnosis of lung adenocarcinoma; wild-type *EGFR* gene; Eastern Cooperative Oncology Group score of 0–2 points; sufficient formalin-fixed, paraffin-embedded samples for *HER-2* mutation detection and DNA sequencing; and patient who did not receive preoperative neoadjuvant therapy. Exclusion criteria were as follows: patients with other tumors or severe diseases, including psychiatric illnesses; and pregnancy. The study protocol was approved by the Ethical Review Committee of the Shanghai Pulmonary Hospital, and the clinical registration number was ChiCTR-OCH-13003507. The study design is outlined in Fig. 1.

### *Detection of* EGFR, anaplastic lymphoma kinase (ALK), ROS proto-oncogene (ROS) 1, and HER-2 mutations

A DNA/RNA Isolation kit (Amoy Diagnostics Co., Xiamen, China) was used according to the manufacturer’s instructions to extract DNA from formalin-fixed, paraffin-embedded tissue samples. Mutations were detected using the Human EGFR Mutation, Human echinoderm microtubule-associated protein-like 4–ALK Fusion Gene, Human ROS1 Fusion Gene, and Human HER-2 Mutation Detection kits (Amoy Diagnostics Co.). PCR amplification conditions were as follows: 95 °C 5 min; 15 cycles of 95 °C for 25 s and 72 °C for 20 s; and 31 cycles of 93 °C for 25 s, 60 °C for 35 s, and 72 °C for 20 s. All samples positive for *HER-2* mutations were confirmed by DNA sequencing using primers with the following sequences: 5'GCC ATG GCT GTG GTT TGT GAT GG3' (forward) and 5'ATC CTA GCC CCT TGT GGA CAT AGG3', which amplified a 342-bp fragment in exon 20 of the *HER-2* gene.

### Immunohistochemistry

HER-2 expression in tissue was detected using a rabbit monoclonal anti-HER-2/Erbb2 (29D8) antibody (diluted 1:200) (Cell Signaling Technology, Danvers, MA, USA). The intensity of the signal was determined as previously described [10], which was as follows: 0, no staining; 1+, < 5 % of cells with strong staining or 5 %–10 % of cells with weak staining; 2+, 5 %–10 % of tumor cells with strong staining or > 10 % of tumor cells with weak staining; and 3+, > 10 % of cells with strong staining. Membranous staining was set as the criteria, and cytoplasmic staining was excluded from the scoring of stain intensity. Scores of 0/1+ and 2+/3+ were counted as negative and positive HER-2 expression, respectively. Samples were independently evaluated by two pathologists.

### Statistical analysis

SPSS v.17.0 software (SPSS Inc., Chicago, IL, USA) was used for statistical analysis. Disease-free survival (DFS) was defined as the time between the start of surgery to the first recorded disease recurrence/metastasis. Overall survival (OS) was defined as the time between the start of surgery to disease-related death. The correlations between *HER-2* mutation and clinical characteristics, as well as between *HER-2* mutation and protein expression, were evaluated with Pearson’s *χ* ^2^ test or Fisher’s exact test. Survival was analyzed with the Kaplan-Meier test, and survival comparisons were carried out with the log-rank test. The two-sided significance level was set at *P* < 0.05.

## Results

### Patient characteristics

Patient characteristics are summarized in Table 1. A total of 1316 formalin-fixed, paraffin-embedded samples from surgical patients at Shanghai Pulmonary Hospital were initially screened, including 1107 cases of lung adenocarcinoma, of which 456 wild-type EGFR lung adenocarcinoma cases were analyzed for *HER-2* mutation. The final sample set included 250 males and 206 females; the median age was 60 years (range: 26–82 years), with 135 and 321 cases ≥ 65 and < 65 years, respectively. In addition, 295 patients had never smoked or were light smokers while 161 were heavy smokers. In terms of disease staging, 262 cases were Stage I, 46 were Stage II, 91 were Stage III, 23 were Stage IV, and 34 were unconfirmed. The 437 cases were classified according to lung adenocarcinoma subtype based on the 2011 criteria. There were 40 cases of lepidic predominant adenocarcinoma (LPA), 187 of acinar predominant adenocarcinoma (APA), 100 of papillary predominant adenocarcinoma (PPA), 35 of solid predominant adenocarcinoma (SPA), 31 of invasive mucinous adenocarcinoma (IMA), 14 of adenocarcinoma in situ (AIS), 14 of minimally invasive adenocarcinoma (MIA), 14 of micropapillary predominant adenocarcinoma (MPA), and two of enteric adenocarcinoma (EA).

**Table 1:** Characteristics of patients with wild-type or mutant *HER-2* and positive or negative HER-2 expression by immunohistochemistry

		HER-2 WT n(%)	HER-2 MUT n(%)	*p* value	HER-2 IHC(-) n(%)	HER-2 IHC(+) n(%)	*p* value
Sex	Male	239(55.1)	11(50.0)	0.666	162(53.6)	30(54.5)	1.000
	Female	195(44.9)	11(50.0)		140(46.4)	25(45.5)	
Age	≥65	132(30.4)	3(13.6)	0.100	80(26.5)	21(38.2)	0.103
	<65	302(69.6)	19(86.4)		222(73.5)	34(61.8)	
Smoking status	Never/light smoking	280(64.5)	15(68.2)	0.822	190(62.9)	40(72.7)	0.172
	Smoking	154(35.5)	7(31.8)		112(37.1)	15(27.3)	
Disease stage	I	249(62.3)	13(59.1)	0.619	184(66.9)	31(62.0)	0.887
	II	43(10.8)	3(13.6)		27(9.8)	5(10.0)	
	III	85(21.3)	6(27.3)		52(18.9)	12(24.0)	
	IV	23(5.8)	0(0.0)		12(4.4)	2(4.0)	
Histologic subtype	Lepidic	40(9.6)	0(0.0)	0.436	34(11.5)	3(5.8)	0.166
	Acinar	179(43.1)	8(36.4)		126(42.7)	20(38.5)	
	Papillary	92(22.2)	8(36.4)		57(19.3)	12(23.1)	
	Micropapillary	14(3.4)	0(0.0)		12(4.1)	1(1.9)	
	Solid	33(8.0)	2(9.1)		20(6.8)	8(15.4)	
	IMA	28(6.7)	3(13.6)		24(8.1)	4(7.7)	
	Enteric	2(0.5)	0(0.0)		1(0.3)	1(0.2)	
	AIS	14(3.4)	0(0.0)		12(4.1)	0(0.0)	
	MIA	13(3.1)	1(4.5)		9(3.1)	3(5.8)	

The total number of wild-type and mutant *HER-2* cases was 456; disease stage and histologic subtype data were available for 422 and 437 of these cases, respectively

The total number of cases negative or positive for HER-2 by immunohistochemistry was 357; disease stage and histologic subtype data were available for 325 and 347 cases, respectively

*Abbreviations*: *AIS* adenocarcinoma in situ, *IMA* invasive mucinous adenocarcinoma, *MIA* minimally invasive adenocarcinoma

### HER-2 *mutation rate*

**Fig. 2:**
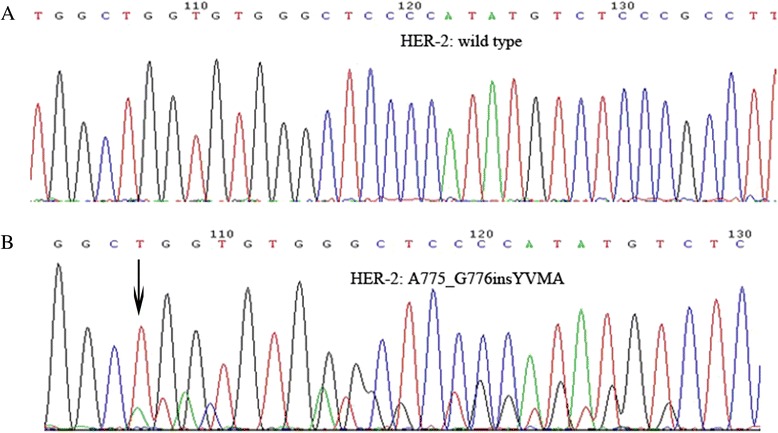
The confirmed results of HER-2 mutations by DNA sequencing. **a** HER-2 wild type; **b** The arrow showed HER-2 A755_G776insYVMA

ARMS analysis was used to detect *HER-2* exon 20 mutations in 456 cases of wild-type EGFR lung adenocarcinoma; 22 (4.8 %) were found to be positive. The presence of an *echinoderm microtubule-associated protein-like 4*/*anaplastic lymphoma kinase* (*ALK*)–*ROS proto-oncogene* (*ROS*) *1* fusion gene was also examined in 390 cases; 331 were found to harbor wild-type *ALK*/*ROS1*. The *HER-2* mutation rate in patients with wild-type EGFR/ALK/ROS1 (i.e., triple-negative samples) was 6.7 %; the 22 samples were analyzed by PCR combined with direct sequencing. For three of the samples, sequencing failed due to insufficient sample quantity. Results for the remaining 19 samples were consistent with those obtained by ARMS: 13 cases were 2324_2325 ins12 (atacgtgatggc), three were 2326_2327insTGT, two were 2325_2326 ins12 (tacgtgatggct), and one was 2331_2332 ins9 (ggctcccca) (Fig. 2).

### Analysis of HER-2 protein expression

**Fig. 3:**
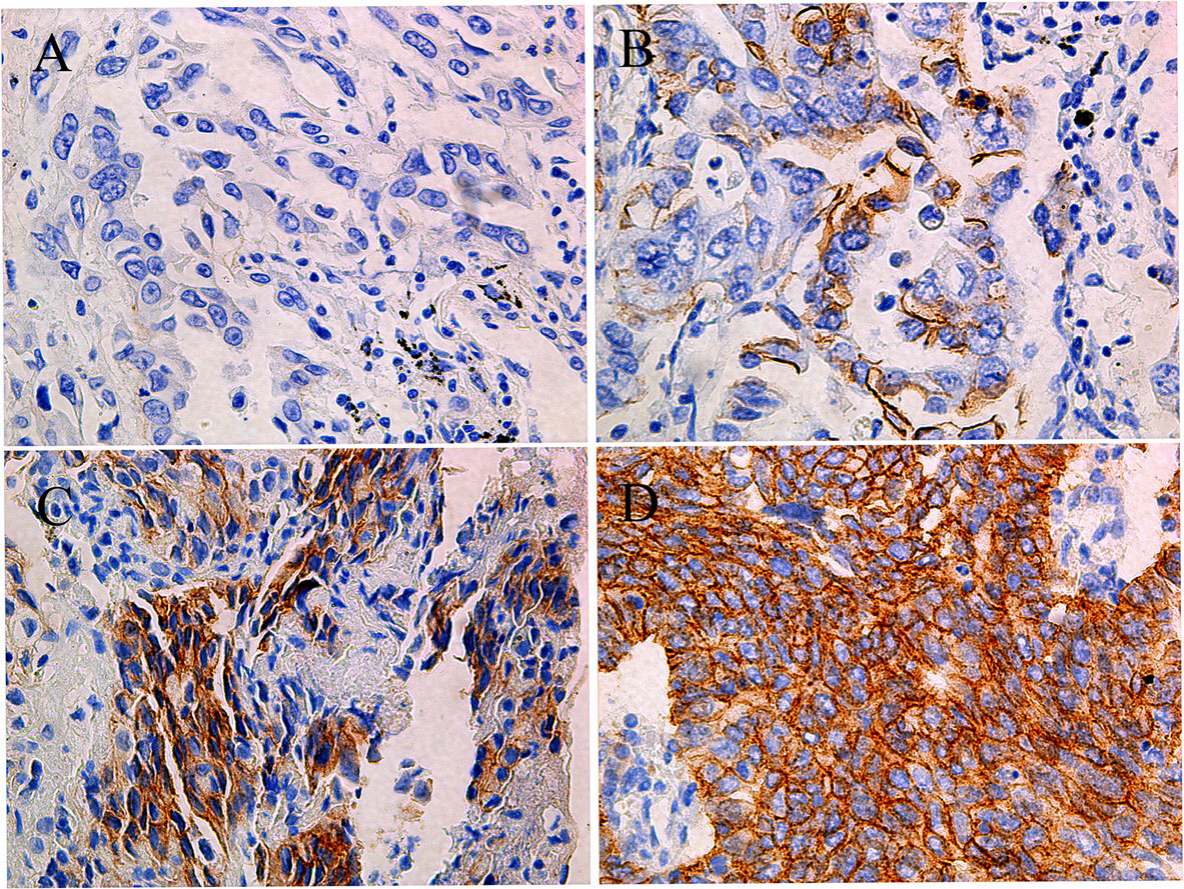
HER-2 protein expression in EGFR wild type lung adenocarcinoma patients by immunohistochemistry: A.0 B.1+ C.2+ D.3+

HER-2 protein expression was assessed in 357 samples by immunohistochemistry. Results were interpreted as previously described [10]. There were 55 positive samples (15.4 %), of which 43 were 2+ (12.0 %) and 12 were 3+ (3.4 %) (Fig. 3).

### *Relationship between* HER-2 *mutation and protein expression and clinical characteristics*

**Fig. 4:**
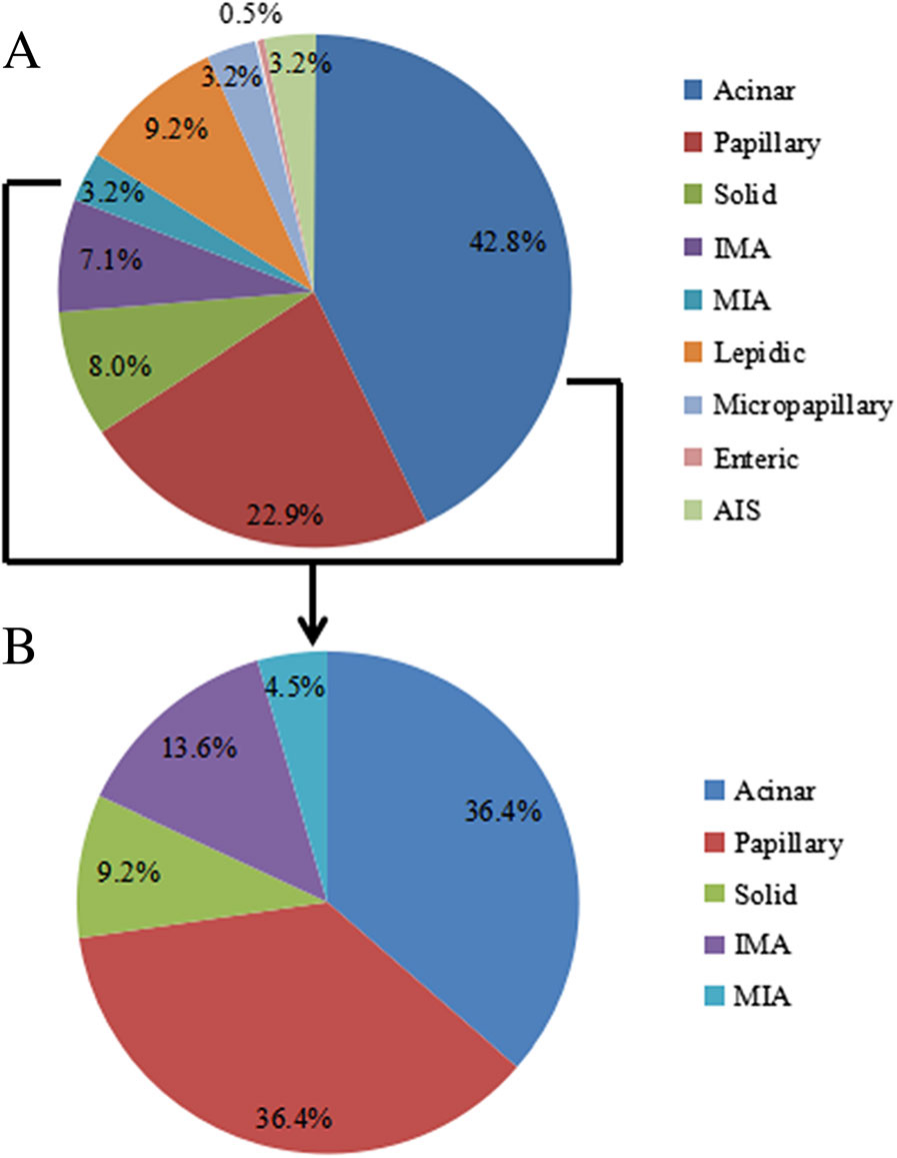
Histologic subtype distribution of the 437 EGFR wild type and 22 HER-2 mutation samples. **a** Acinar and papillary are the most common histologic subtypes in lung adenocarcinoma; **b** HER-2 mutations occurred in acinar, papillary, solid, IMA, and MIA subtypes, other subtypes were not found (IMA, invasive mucinous adenocarcinoma; MIA, minimally invasive adenocarcinoma)

The presence of *HER-2* mutations was not correlated with clinical characteristics, including gender, age, smoking status, histological subtype, and disease stage. However, the mutation rate differed among lung adenocarcinoma subtypes: the 22 cases of *HER-2* mutation were observed in APA (36.4 %), PPA (36.4 %), MIA (13.6 %), SPA (9.2 %), and IMA (4.5 %), whereas no mutations were detected in LPA, MPA, EA, or AIS (Table 1 and Fig. 4). Furthermore, there was no correlation between *HER-2* mutation and protein expression. Simultaneous *HER-2* mutation and HER-2 overexpression was observed in only two samples.

### Relationship between prognosis and HER-2 mutation or protein expression

**Fig. 5:**
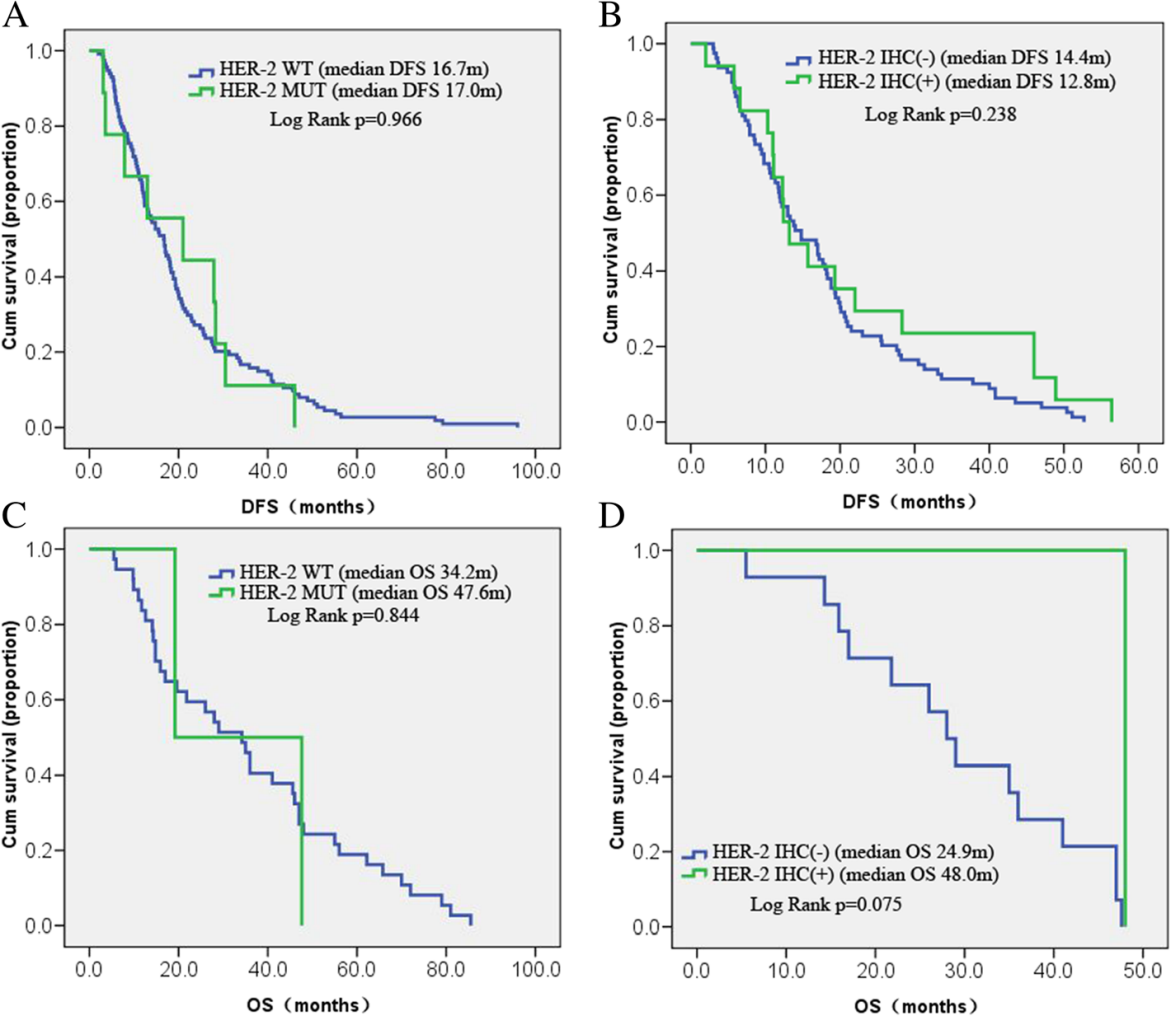
DFS and OS were analyzed according to HER-2 mutations status and protein expression level, respectively. **a** DFS of HER-2 WT (*n* = 114) and HER-2 MUT (*n* = 9) patients; **b** DFS of HER-2 IHC (-) (*n* = 79) and HER-2 IHC (+) (*n* = 17) patients; C. OS of HER-2 WT (*n* = 37) and HER-2 MUT (*n* = 2) patients; D. OS of HER-2 IHC (-) (*n* = 14) and HER-2 IHC (+) (*n* = 1) patients. WT: wild type group, MUT: mutation group; and HER-2 IHC (-): the score 0 or 1; IHC (+): The score 2 or 3

At the last follow-up, 123 patients achieved DFS, and OS data were collected from 39 patients. Most of the remaining patients continued to survive, and follow-up data are needed. A subset of patients was lost to follow-up. *HER-2* mutation was not associated with DFS (median: 16.7 vs. 17.0 months; P = 0.966). The difference with OS was also not statistically significant (median: 34.2 vs. 47.6 months; P = 0.844). DFS data were obtained from 79 and 17 patients who were negative and positive for HER-2, respectively, by immunohistochemistry. However, DFS did not differ significantly between the two groups (median: 14.4 vs. 12.8 months, P = 0.238). The 14 HER-2-negative and one HER-2-positive patients had similar OS (median: 24.9 vs. 48.0 months, P = 0.075) (Fig. 5). We also analyzed the DFS data of the HER2 IHC(-) versus HER2 IHC (+) in stage I/II and stage III/IV cases, respectively (Additional file 1: Figure S1) and found that none of them had a significantly difference.

## Discussion

In the present study, *HER-2* mutation rate and protein expression level were measured in 456 patients with wild-type EGFR lung adenocarcinoma. The *HER-2* mutation rate was 4.8 % while the rate in EGFR/ALK/ROS1 triple-negative samples was 6.7 %. There was no correlation between *HER-2* mutation and clinical characteristics. The rate differed according to lung adenocarcinoma subtype and was highest in APA (36.4 %) and PPA (36.4 %).

*HER-2* mutations have been linked to NSCLC. Other studies have shown that they also occur in lung adenocarcinoma, with mutation rates from 2 % up to 14.1 % [9, 10, 17]. Our results were consistent with previous studies reporting a *HER-2* mutation rate of 1.98 % in the overall population, 4.8 % in wild-type EGFR cases, and 6.7 % in EGFR/ALK/ROS1 triple-negative cases. The most commonly observed *HER-2* mutation is an in-frame insertion in exon 20 [6]. An analysis of *HER-2* mutations in exons 18–21 identified cases harboring the same exon 20 insertion [10]. In the present study, DNA sequencing of 19 patients revealed 15 cases with A775_G776ins12YVMA, three with 2326_2327insTGT, and one with 2331_2332 ins9 (ggctcccca), all of which are in exon 20. HER-2 inhibitors such as trastuzumab and afatinib were effective in inhibiting exon 20 insertion mutations [18, 19], which are thus a useful biomarker for assessing the therapeutic efficacy of these inhibitors.

*HER-2* mutations are more common in female non-smokers with moderately to poorly differentiated adenocarcinoma, small tumor load, and early-stage lung cancer, but are not linked to patient prognosis [10, 11, 17]. Two studies in the Chinese population also found that *HER-2* mutations were prevalent in female non-smokers, but were not correlated with prognosis [10, 20], while a study from Taiwan found an association between *HER-2* mutation and late stages of disease [8]. We did not observe a correlation between mutation status and patient characteristics such as gender, age, disease stage, smoking status, or prognosis.

APA and PPA subtypes of lung adenocarcinoma accounted for 65.7 % of all cases examined in this study. These results are consistent with those reported by others; for instance, among Caucasians, *HER-2* mutations were primarily observed in PPA, APA, SPA, and MPA [9], whereas another study found that mutation rates were higher in PPA, AIS/MPA, LPA, and SPA [11]. Thus, *HER-2* mutations are mainly associated with APA, PPA, IMA, SPA, and MIA subtypes with some similarity across ethnic groups; HER-2 inhibitors may be effective in such patients. Therefore, more emphasis should be placed on the classification of lung adenocarcinoma histological subtypes and *HER-2* mutation analysis in clinical settings.

## Conclusions

In summary, this study showed that HER-2 mutation was not correlated to HER-2 protein overexpression, and existing follow-up data did not show a correlation between HER-2 mutation with DFS or OS.

## Additional file

Additional file 1: Figure S1. DFS of HER-2 IHC (-)/(+) patients in stage I/II and III/IV. A. DFS of HER-2 IHC (-) (*n* = 45) and IHC (+) (*n* = 10) patients in stage I/II. B. DFS of IHC (-) (*n* = 31) and IHC (+) (*n* = 6) patients in stage III/IV. (TIF 1145 kb)

## Abbreviations

AIS: Adenocarcinoma in situ
ALK: Anaplastic lymphoma kinase
APA: Acinar predominant adenocarcinoma
ARMS: Amplification-refractory mutation system
DFS: Disease-free survival
EA: Enteric adenocarcinoma
EGFR: Epidermal growth factor receptor
HER-2: Human epidermal growth factor receptor-2
IMA: Invasive mucinous adenocarcinoma
LPA: Lepidic predominant adenocarcinoma
MIA: Minimally invasive adenocarcinoma
MPA: Micropapillary predominant adenocarcinoma
NSCLC: Non-small cell lung cancer
OS: Overall survival
PPA: Papillary predominant adenocarcinoma
ROS1: ROS proto-oncogene 1
SPA: Solid predominant adenocarcinoma
