# Supplementary figures and images for: Epidemiological study of HER-2 mutations among EGFR wild-type lung adenocarcinoma patients in China
Source: BMC Cancer. 2016 Oct 28;16:828. doi: 10.1186/s12885-016-2875-z (PMC5084329; doi:10.1186/s12885-016-2875-z)

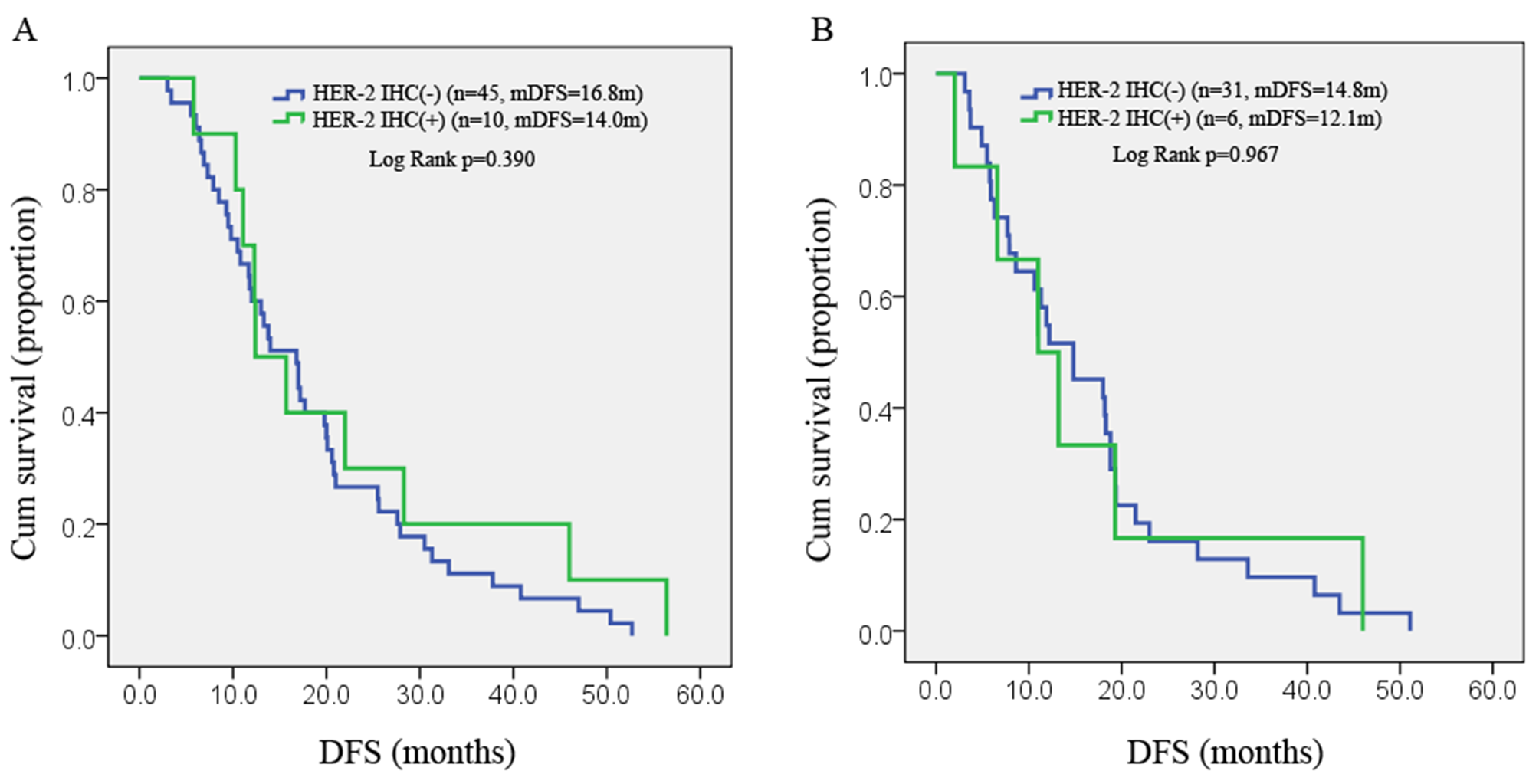

Supplement: Additional file 1: Figure S1. — DFS of HER-2 IHC (-)/(+) patients in stage I/II and III/IV. A. DFS of HER-2 IHC (-) (n = 45) and IHC (+) (n = 10) patients in stage I/II. B. DFS of IHC (-) (n = 31) and IHC (+) (n = 6) patients in stage III/IV. (TIF 1145 kb) [file 12885_2016_2875_MOESM1_ESM.tif]
